# Autoimmune Hepatitis-like Syndrome in a Patient with Ankylosing Spondylitis: A Case Report

**DOI:** 10.3390/reports9020143

**Published:** 2026-05-04

**Authors:** Nicoleta Maria Crăciun Ciorba, Ilie Marius Ciorba

**Affiliations:** 1 Department of Internal Medicine II, Faculty of Medicine, George Emil Palade University of Medicine, Pharmacy, Science and Technology of Târgu Mureș, 540139 Târgu Mureș, Romania; 2 Internal Medicine I Clinic, County Emergency Clinical Hospital of Târgu-Mureș, 540136 Târgu Mureș, Romania; 3 Department of Internal Medicine I, Faculty of Medicine, George Emil Palade University of Medicine, Pharmacy, Science and Technology of Târgu Mureș, 540139 Târgu Mureș, Romania; 4 Gastroenterology Clinic, County Emergency Clinical Hospital of Târgu-Mureș, 540136 Târgu Mureș, Romania

**Keywords:** autoimmune hepatitis, ankylosing spondylitis, axial spondyloarthritis, anti-LC-1, autoimmune liver disease, case report

## Abstract

**Background and clinical significance**: Autoimmune hepatitis (AIH) and ankylosing spondylitis (AS) are distinct immune-mediated disorders that only rarely coexist. Diagnostic interpretation becomes especially challenging when the liver biochemistry is not classically hepatocellular and the histology is unavailable. **Case presentation**: We report a 51-year-old man with inflammatory back pain, polyarthralgia, weight loss, fatigue, night sweats and fever. Laboratory tests showed marked systemic inflammation, anemia and a cholestatic-predominant liver profile with associated aminotransferase elevation. Imaging demonstrated bilateral sacroiliitis and syndesmophytosis. Liver workup excluded viral, obstructive, metabolic, hereditary and inflammatory bowel disease-associated cholangiopathic causes. Antinuclear antiboidies (ANA) and anti liver cyotsole 1 antiboidies (anti-LC-1) were positive, IgG was mildly elevated, magnetic resonance cholangio-pancreatography (MRCP) was negative for primary sclerosing cholangitis and the simplified AIH score was six. A liver biopsy was proposed but refused. The patient received a short course of prednisone for rheumatologic flare control, followed by nonsteroidal anti-inflammatory treatment and sulfasalazine, with normalization of liver tests during follow-up. **Conclusions**: This case is suggestive, but not diagnostic, of autoimmune hepatitis in a patient with ankylosing spondylitis. In the absence of histology and in the setting of a cholestatic-predominant biochemical profile, the findings may be more appropriately interpreted as an autoimmune hepatitis-like syndrome. The main teaching point is that abnormal liver tests in AS warrant structured evaluation beyond drug toxicity and viral hepatitis, particularly when autoimmune serology is positive, even in a cholestatic-predominant presentation.

## 1. Introduction and Clinical Significance

Ankylosing spondylitis (AS) is a chronic inflammatory disease that primarily affects the axial skeleton, characterized by progressive spinal inflammation that may lead to vertebral fusion, reduced mobility and the development of abnormal postures [1,2,3]. The disease typically manifests in early adulthood, most commonly around the age of 30, and shows a higher prevalence in males [1]. AS is a systemic condition, with extra-articular involvement frequently observed at musculoskeletal, ocular, cardiovascular, pulmonary and gastrointestinal levels [2,3]. Immune-mediated diseases such as ankylosing spondylitis, psoriasis and primary sclerosing cholangitis are commonly reported in patients with inflammatory bowel disease (IBD) [4,5,6]. Extraintestinal manifestations occur in a significant proportion of IBD patients, with musculoskeletal involvement being among the most frequent [5]. Although hepatobiliary manifestations are well recognized in IBD, autoimmune hepatitis (AIH) remains a relatively rare association, with a reported prevalence of approximately 2–8% according to the available literature [7,8]. Autoimmune hepatitis and ankylosing spondylitis are distinct autoimmune disorders that, in rare instances, may coexist in the same patient [9]. Their concurrent presentation has raised interest regarding potential shared pathogenic pathways that may predispose individuals to the development of multiple autoimmune conditions [10,11]. AIH is characterized by chronic immune-mediated inflammation directed against hepatocytes [7,12], while AS predominantly involves the inflammation of the sacroiliac joints and axial skeleton [3,13]. Despite differences in their primary target organs, both conditions exhibit features that are typical of systemic autoimmunity, including genetic susceptibility, dysregulation of immune tolerance and chronic inflammatory pathways involving cytokines and human leukocyte antigen (HLA) associations [3,10]. The coexistence of AIH and AS presents diagnostic and therapeutic challenges, particularly when one condition develops in a patient with an established autoimmune disease, as immunosuppressive treatment strategies may influence disease activity at multiple organ levels [9,12]. Understanding potential overlapping immunological mechanisms remains an important area of clinical and research interest, as it may influence disease recognition, monitoring strategies and therapeutic decision-making. The present case is noteworthy because a liver disease process that was suggestive of autoimmune hepatitis was identified in a patient with ankylosing spondylitis, despite a cholestatic-predominant biochemical profile, negative MRCP, absence of inflammatory bowel disease, and unavailable histology after biopsy refusal.

## 2. Case Presentation

A 51-year-old man with hypertension and no other significant past medical history presented with polyarthralgia. He reported a 10-year history of inflammatory low back pain and limited spinal mobility, followed by a 3-month period of asthenia, fatigue, dry cough, night sweats and fever, together with a 20 kg weight loss. He denied alcohol use and the use of prescription, over-the-counter, or recreational drugs other than antihypertensive treatment (perindopril, indapamide and amlodipine). He also denied the use of herbal supplements or other potentially hepatotoxic exposures. There was no family history of autoimmune or liver disease. On examination, the patient was pale and had diffuse spinal tenderness to percussion, with painful limitation of lower-limb movement. The remainder of the examination was unremarkable.

Laboratory testing showed elevated inflammatory markers (ESR and CRP), severe anemia, thrombocytosis, elevated liver enzymes and cholestasis. Using the R value (R = (ALT/ULN)/(ALP/ULN)) to characterize the liver injury pattern, the admission values yielded R ≈ (109.6/41)/(417/130) ≈ 0.83, which was consistent with a cholestatic-predominant biochemical pattern (R < 2) despite concomitant aminotransferase elevation. Accordingly, we interpreted the profile as being cholestatic-predominant with associated hepatocellular injury, rather than as a purely hepatocellular pattern.

Given the presence of anemia, elevated inflammatory markers and aminotransferase and cholestatic enzyme elevations (Table 1), an abdominal ultrasound was performed and excluded biliary obstruction, stenosis, and focal liver lesions.

Additional screening was performed to exclude viral hepatitis (hepatitis A IgM, hepatitis B surface and e antigens and anti-hepatitis C antibodies, all negative) and hereditary or metabolic disorders (including ferritin, urinary copper and alpha-1 antitrypsin deficiency testing), all of which were within normal ranges. HLA-B27 testing was positive. Given the elevated liver enzymes, we performed an autoimmune liver disease panel, which showed positive antinuclear antibodies (ANA) at ≥1:160 and positive anti-liver cytosol type 1 (anti-LC-1) antibodies. The results were negative for the antimitochondrial antibody fractions M2-3E, sp100, gp210 and promyelocytic leukemia protein (PML), as well as for anti-liver kidney microsome type 1 (LKM-1), anti-soluble liver antigen/liver-pancreas (SLA/LP), anti-actin and anti-Ro-52 antibodies. MRCP showed no lesions suggestive of primary sclerosing cholangitis. Liver biopsy was discussed but declined by the patient. Accordingly, AIH remained a probable rather than definitive diagnosis: the simplified autoimmune hepatitis score was six, but histologic confirmation could not be obtained.

Serum protein electrophoresis was performed with the following values: Alpha/Gamma: 0.75, Albumin: 42.9%, Alpha1: 6.9%, Alpha 2: 17.4%, Beta: 17.1, and Gamma: 15.7% This revealed an elevated gamma fraction. Immunoglobulin profiling showed IgA 283 mg/dL, IgG 1825 mg/dL (laboratory ULN 1600 mg/dL) and IgM 140 mg/dL, confirming mildly elevated IgG.

Simplified AIH score components (simplified IAIHG score reported as six; histology unavailable):

- Autoantibodies (2 points): ANA ≥1:160 and anti-LC-1 positive.
- IgG (2 points): IgG 1825 mg/dL (>1.1 × ULN; laboratory ULN, 1600 mg/dL).
- Viral hepatitis excluded (2 points): HAV IgM negative, HBsAg/HBeAg negative, anti-HCV negative.
- Histology (0 points): Liver biopsy discussed but refused; no histological scoring component.

To exclude malignancy, we measured alpha-fetoprotein, carbohydrate antigen 19-9, carcinoembryonic antigen and prostate-specific antigen, all of which were within normal ranges.

Given the association between ankylosing spondylitis and inflammatory bowel disease (IBD), fecal calprotectin was measured despite the absence of gastrointestinal symptoms and was 71 µg/mL (cutoff 50 µg/mL). Upper gastrointestinal endoscopy and colonoscopy were therefore performed with no macroscopic lesions identified, and the biopsies were also negative for IBD. Although enteroscopy was unavailable, abdominal MRI of the small bowel showed no evidence of inflammation or other findings suggestive of inflammatory bowel disease.

**Figure 1:**
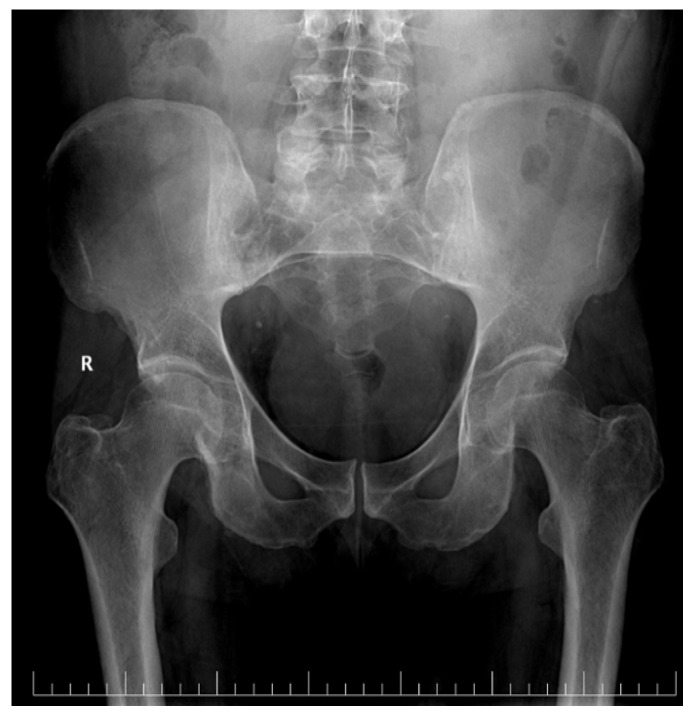
Pelvic X-ray showing narrowing of the right coxofemural joint space.

X-rays of the cranium and pelvis were also performed (Figure 1), which highlighted a narrowing of the right coxofemural joint space but no osteolytic lesions.

**Figure 2:**
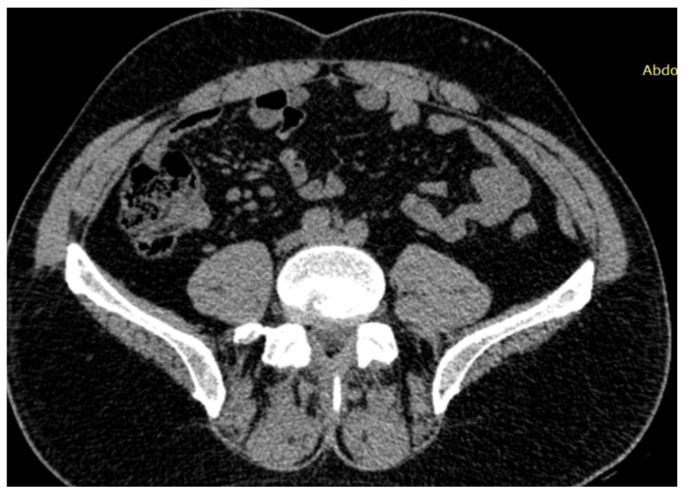
CT showing clustered mesenteric lymph nodes with inflammatory features.

**Figure 3:**
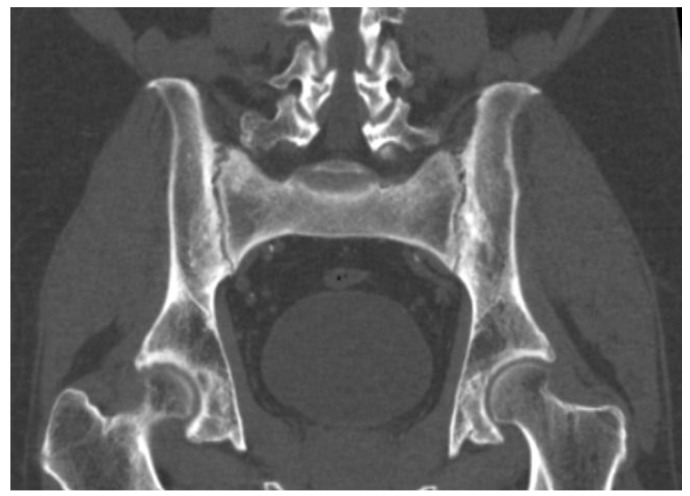
CT showing arthritic changes in the sacroiliac joints.

Whole-body computed tomography showed multiple mesenteric lymph nodes with inflammatory features (Figure 2), syndesmophytes at the T3–T5 and T12–L1 levels and arthritic changes in the sacroiliac joints (Figure 3).

**Figure 4:**
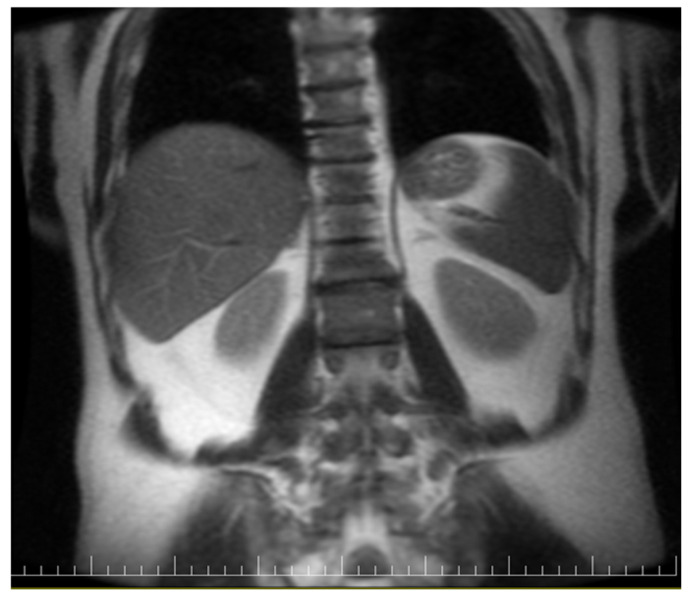
Syndesmophytes on imaging.

**Figure 5:**
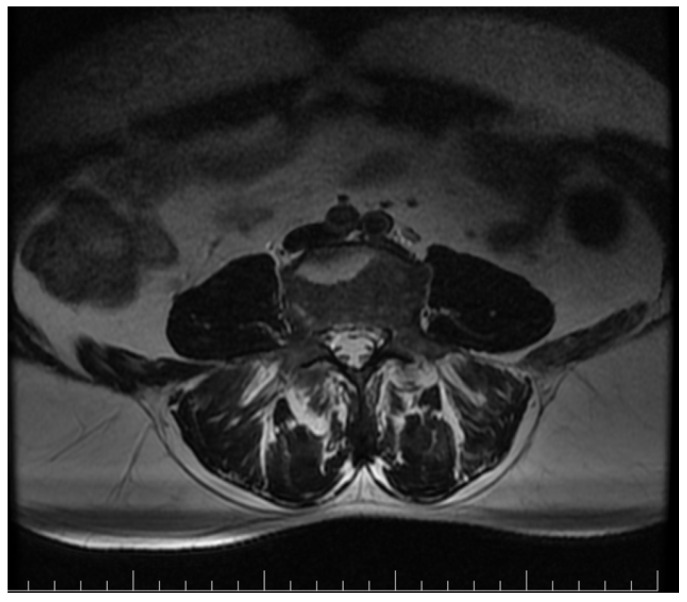
MRI showing inflammatory sacroiliac changes, including a geode-like subchondral cystic lesion and subchondral cystic change in the right acetabulum.

Pelvic MRI showed inflammatory changes in the sacroiliac joints, including erosions and subchondral bone marrow edema, predominantly on the right side, and confirmed the previously described syndesmophytes (Figure 4). These lesions were hyperintense on T2-weighted and STIR sequences and included a geode-like subchondral cystic lesion and subchondral cystic change in the right acetabulum (Figure 5).

Because the patient remained completely asymptomatic from a gastrointestinal standpoint and the upper gastrointestinal endoscopy and colonoscopy did not reveal relevant lesions, a short course of corticosteroid therapy (prednisone 0.5 mg/kg/day for 5 days) was given upon rheumatologic recommendation for flare control. During this interval, the liver parameters improved, but the pain remained severe (NRS 9) and CRP remained elevated. Nonsteroidal anti-inflammatory treatment was therefore introduced after metamizole 500 mg twice daily and the short prednisone course proved insufficient for symptom control. Both regimens were well tolerated and blood glucose and blood pressure were carefully monitored. A rheumatology consultation established the diagnosis of mixed ankylosing spondyloarthritis with bilateral stage 2 sacroiliitis and recommended sulfasalazine together with NSAIDs as needed.

Sulfasalazine was started at 500 mg/day for the first week and then increased by 500 mg/day each week to a final dose of 4 g/day. The complete blood count and liver-function monitoring plan began with the baseline values obtained before treatment initiation, followed by monitoring of the complete blood count with differential and platelet count, ALT, AST, ALP, GGT, total and direct bilirubin, albumin and creatinine every 2 weeks for the first 6 weeks, monthly until 3 months and every 3 months thereafter while the patient remained on a stable dose. Silymarin and essential amino acids were also prescribed. Because sulfasalazine initiation, supportive treatment and spontaneous biochemical improvement overlapped in time, the subsequent normalization of liver tests should be interpreted as a favorable clinical evolution, rather than as proof of a specific etiologic diagnosis or liver-directed treatment effect. At the 4-week follow-up, the liver enzymes had returned to normal. During follow-up, the patient reported taking the prescribed treatment as directed, including the sulfasalazine up-titration, and also complete relief of symptoms and no treatment-related adverse effects. He considered the overall outcome favorable and agreed with the continued clinical and biochemical monitoring. At 3-month follow-up, he remained asymptomatic and the bloodwork showed normal inflammatory markers and a normal liver profile (Table 2).

**Table 2:** Timeline of clinical course.

Timepoint	Key Clinical Findings and Investigations	Management and Outcome
~10 years prior	Chronic lumbar pain and limitation of spinal mobility began (history).	No specific treatment documented.
February 2024	Several evaluations for polyarthralgia (per history).	-
~3 months before admission	Insidious onset of polyarthralgia, fatigue/asthenia, dry cough, night sweats; ~20 kg weight loss over ~2 months.	-
2 days before admission	Fever for two days prior to ED presentation.	-
Admission (inpatient day 0)	Exam: pallor; painful spine on percussion; limited/painful lower limb movement. Labs: markedly elevated inflammatory markers; severe anemia; thrombocytosis; mild transaminase elevation and cholestasis (Table 1). Abdominal ultrasound: no biliary obstruction or focal liver lesions.	Diagnostic workup initiated.
Inpatient evaluation	Viral hepatitis excluded (HAV IgM, HBsAg/HBeAg, anti-HCV negative). Metabolic/hereditary testing normal (ferritin, urinary copper, A1AT). HLA-B27 positive. Autoimmune liver profile: ANA and anti-LC-1 positive; other liver autoantibodies negative. MRCP negative for PSC. SPEP: elevated gamma fraction; IgG 1825 mg/dL. Tumor markers (AFP, CA19-9, CEA, PSA) normal. GI screening: fecal calprotectin 71 µg/mL; EGD/colonoscopy with biopsies negative for IBD; small bowel MRI negative. Imaging: pelvic X-ray (right hip joint space narrowing); CT shows mesenteric inflammatory nodes + syndesmophytes and sacroiliac changes; pelvic MRI shows sacroiliitis with erosions and bone marrow edema + syndesmophytes.	Short-course prednisone 0.5 mg/kg/day for 5 days had been given for flare control and coincided with initial biochemical improvement, but pain (NRS 9) and CRP remained high. NSAIDs were then introduced. Rheumatology consult: mixed ankylosing spondyloarthritis; bilateral stage 2 sacroiliitis; recommends sulfasalazine and NSAIDs PRN. Liver biopsy discussed but refused.
Discharge/treatment plan	Inflammatory markers and liver tests improving by discharge (Table 1).	Sulfasalazine started 500 mg/day with weekly +500 mg titration to 4 g/day. Silymarin and essential amino acids prescribed. Because several interventions overlapped, subsequent normalization of liver tests was interpreted cautiously and not attributed to a single therapy.
4-week follow-up	Liver enzymes normalized.	Symptoms completely alleviated. No adverse effects observed. Favorable biochemical follow-up maintained.
3-month follow-up	Normal inflammatory markers and normal liver profile; asymptomatic.	Continued favorable outcome.

## 3. Discussion

Patients who exhibit features of both autoimmune hepatitis (AIH) and ankylosing spondylitis (AS) may present with a complex clinical picture in which liver-test abnormalities risk being attributed too narrowly to systemic inflammation, medication exposure or a single specialty-specific diagnosis [7,8,11]. Symptom onset may be temporally related or separated by years, but in either scenario, early manifestations can be subtle enough to delay the recognition of concomitant immune-mediated disease [8].

Our patient had no overt liver-related symptoms, but liver tests showed a cholestatic-predominant pattern with concomitant hepatocellular injury, which appropriately broadened the differential diagnosis. After the exclusion of viral and metabolic causes and identification of ANA and anti-LC-1 positivity with mildly elevated IgG, the findings were considered to be suggestive of autoimmune hepatitis; however, they were not diagnostic of classical AIH. Although the simplified AIH score was six, the histology was unavailable because liver biopsy was refused, and the biochemical pattern was not classically hepatocellular. For this reason, the case may be more appropriately framed as an AIH-like syndrome or immune-mediated liver disease suggestive of AIH, rather than definitive or classical AIH [7,12,14].

The cholestatic-predominant profile lowers diagnostic certainty and requires an explicit discussion of alternatives. MRCP did not support primary sclerosing cholangitis, colonoscopy with biopsies and small-bowel MRI did not support inflammatory bowel disease and PBC-specific antibodies were negative, making PSC, PBC and overt cholestatic overlap syndromes less likely. However, overlap syndromes could not be excluded completely without liver histology. Likewise, inflammation-associated cholestasis or another immune-mediated hepatobiliary process remains possible. There was no history of alcohol misuse, herbal exposure or obvious hepatotoxic medication use beyond chronic antihypertensive treatment, so drug-induced liver injury was not favored. Nevertheless, inflammation-associated cholestasis or another AIH-like immune liver process cannot be excluded completely in the absence of biopsy.

An additional source of uncertainty is the use of corticosteroids before the diagnostic clarification was complete. The patient received prednisone 0.5 mg/kg/day for 5 days for rheumatologic flare control and this coincided with the initial biochemical improvement. Even a short course of corticosteroids may reduce aminotransferases and cholestatic enzymes, attenuate hepatic inflammation and temporarily improve immune-mediated liver disease activity. Accordingly, the observed laboratory improvement cannot be interpreted as specific evidence supporting AIH, because corticosteroid exposure may have partially treated an underlying inflammatory liver process while simultaneously obscuring the diagnostic clarity. Subsequent normalization of liver tests must therefore be interpreted cautiously, particularly because prednisone, NSAIDs, sulfasalazine, supportive treatment and possible spontaneous improvement overlapped in time. Current guidance continues to place liver histology at the center of diagnostic confirmation, exclusion of overlap syndromes, and disease staging. In this patient, long-term AIH-directed therapy was not initiated on the basis of a firm hepatologic diagnosis. Therefore, the favorable biochemical evolution is clinically reassuring but should not be interpreted as proof of classical AIH, proof of treatment response specific to AIH, or proof against alternative immune-mediated or cholestatic processes [7,15].

## 4. Limitations

This report has several important limitations. First, diagnostic uncertainty remains substantial because liver biopsy was not performed. As a result, the diagnosis cannot be histologically confirmed, disease staging cannot be assessed and typical interface hepatitis or alternative pathological patterns could not be evaluated. Second, the biochemical profile was cholestatic-predominant rather than classically hepatocellular, which reduces confidence in labeling the presentation as classical autoimmune hepatitis. Third, overlap syndromes and other immune-mediated cholestatic or hepatitic processes cannot be fully excluded in the absence of histology, even though MRCP, serology and endoscopic evaluation reduced the likelihood of several major alternatives. Fourth, short-course corticosteroid exposure occurred before diagnostic clarification was complete and may have improved the liver biochemistry, thereby acting as a confounding factor. Finally, the subsequent normalization of liver tests occurred in the setting of overlapping interventions and follow-up, rather than under a clearly defined AIH-specific treatment strategy, which limits causal interpretation.

## 5. Conclusions

This case describes a liver disease presentation that is suggestive, but not diagnostic, of autoimmune hepatitis in a patient with ankylosing spondylitis. The interpretation is supported by positive ANA and anti-LC-1 antibodies, mildly elevated IgG, exclusion of several competing causes of liver disease, and a simplified AIH score of six, but remains limited by the absence of histology, the cholestatic-predominant biochemical pattern, and corticosteroid exposure before full diagnostic clarification. The main clinical message is that abnormal liver tests in ankylosing spondylitis require a structured evaluation beyond drug toxicity and viral hepatitis, while diagnostic conclusions should remain cautious when histologic confirmation is unavailable.

## Figures and Tables

**Table 1:** Bloodwork evolution during inpatient stay.

Parameter	On Admission	Midway Evaluation	On Discharge	Reference Range	× ULN (On Admission)
WBC ×10^3^/µL	11.4	10.2	9.2	4–10 × 10^3^/µL	1.14
Hemoglobin g/L	75	94	97.2	130–170 g/L	0.44
Hematocrit %	21.9	29.4	29.5	40–52%	0.42
Platelets ×10^3^/µL	638	659	576	150–400 × 10^3^/µL	1.59
INR	1.02	0.98	0.96	0.8–1.2	0
Serum iron (µmol/L)	4.06	10.2	24	10–30 µmol/L	0.14
ESR (mm/h)	105	98	74	0–15 mm/h	7
CRP (mg/L)	235.4	180.5	65.4	0–5 mg/L	47.08
Fibrinogen	635	380	245	200–400 mg/dL	1.59
Serum proteins (g/L)	62.5	69	71	64–83 g/L	0.75
Albumin (g/L)	29.8	30.1	34	35–50 g/L	0.6
AST (U/L)	85.45	25.2	19.3	0–40 U/L	2.14
ALT (U/L)	109.6	58.4	24.5	0–41 U/L	2.67
ALP (U/L)	417	258	179	40–130 U/L	3.21
GGT (U/L)	246	95.2	45	8–61 U/L	4.03

Abbreviations: WBC, white blood cells; INR, international normalized ratio; ESR, erythrocyte sedimentation rate; CRP, C-reactive protein; AST, aspartate aminotransferase; ALT, alanine aminotransferase; ALP, alkaline phosphatase; and GGT, gamma-glutamyl transferase.

## Data Availability

The original contributions presented in this study are included in the article material. Further inquiries can be directed to the corresponding author.

## Abbreviations

The following abbreviations are used in this manuscript:

AIH	Autoimmune hepatitis
AS	Ankylosing spondylitis
IBD	Inflammatory bowel disease
HLA	Human leukocyte antigen
INR	International normalized ratio
ESR	Erythrocyte sedimentation rate
CRP	C-reactive protein
ALT	Alanine aminotransferase
ALP	Alkaline phosphatase
AST	Aspartate aminotransferase
GGT	Gamma-glutamyl transferase
WBC	White blood cells
ANA	Antinuclear antibodies
LC-1	Anti-liver cytosol type 1 antibodies
M2-3E	Antimitochondrial antibody fractions
PML	Promyelocytic leukemia protein
LKM-1	Anti-liver kidney microsome type 1
SLA/LP	Anti-soluble liver antigen/liver–pancreas
MRCP	Magnetic resonance cholangiopancreatography
HAV	Hepatitis A virus
HBsAg	Hepatitis B surface antigen
HBeAg	Hepatitis B e-antigen
HCV	Hepatitis C virus
EGD	Esophagogastroduodenoscopy
MRI	Magnetic resonance imaging
NSAID	Nonsteroidal anti-inflammatory drug
AFP	Alpha-fetoprotein
CA 19-9	Carbohydrate antigen 19-9
CEA	Carcinoembryonic antigen
PSA	Prostate-specific antigen
